# Application of Zinc Oxide to Obtain and Modify Properties of Adipate Plasticizer of Polyvinyl Chloride

**DOI:** 10.3390/polym12081728

**Published:** 2020-08-01

**Authors:** Aliya K. Mazitova, Irina N. Vikhareva, Guliya K Aminova, Juliya N. Savicheva

**Affiliations:** 1 Applied and Natural Sciences Department,,Ufa State Petroleum Technological University, Mendeleeva St. 195, 450080 Ufa, Russia; elenaasf@yandex.ru (A.K.M.); aminovagk@inbox.ru (G.K.A.); 2 Department of Technological machines and equipment, Ufa State Petroleum Technological University, Kosmonavtov St. 1, 450062 Ufa, Russia; ufa.rusoil@gmail.com

**Keywords:** adipate plasticizer, amphoteric catalyst, zinc oxide, ethoxylated alcohol, polyvinyl chloride, esterification

## Abstract

Heterogeneous catalysts are widely used in basic research and in the petrochemical industry due to their effectiveness. In modern times, interest in this type of catalyst and, in particular, in mineral oxides is associated with the technological design of the process, namely: The absence of waste emissions, and the possibility of regeneration and reuse of the catalyst, which meets the criteria of green chemistry. For this reason, the preparation of non-toxic adipate plasticizers is expediently carried out under conditions of heterogeneous catalysis. Zinc oxide was chosen for this purpose, and as initial reagents: Adipic acid, butoxyethanol, and phenoxyethanol. For synthesized butoxyethylphenoxyethyl adipate by thermogravimetry on a TGA-DSC-combined thermal analysis instrument and differential scanning calorimetry on a DSC-1 instrument («Mettler Toledo»), the following properties were studied: Thermal stability, melting and crystallization temperatures, polymer compatibility. An analysis of the data in comparison with di-2-ethylhexylphthalate confirmed the possibility of using it as a plasticizing additive in PVC compositions. The zinc compound obtained in situ as part of the developed plasticizing composition contributes to increasing the color and thermal stability of the obtained PVC compositions.

## 1. Introduction

Polyvinyl chloride (PVC) is one of the most common plastics used today due to its low cost, durability, versatility, and the possibility of modifying properties using various additives [1,2]. Among the most important additives are plasticizers, which account for up to 60% of the plastic compound [3,4,5].

The leading positions in the volume of applications are taken by ester plasticizers, including compounds based on adipic acid [6,7,8]. This type of plasticizer is characterized by low toxicity, biodegradability, and complies with the strict environmental and safety rules formulated in the Rio Declaration on environment and development [9,10,11,12,13].

The main way to obtain adipic acid esters is esterification [14,15]. The catalysis of this process is carried out using homogeneous catalysts, for example, mineral acids (H_2_SO_4_, HF, H_3_PO_4_), organic sulfonic acids (paratoluene sulfonic acid, benzenesulfonic acid), or using heterogeneous catalysts (metal oxides and hydroxides, salts, zeolites, clays).

The use of acidic catalysts negatively affects the quality of the resulting ester, leads to the formation of by-products, causes problems in the isolation and purification, and is also characterized by the inability to reuse the catalyst, the need for its disposal, and corrosion of the equipment [16].

The growing demand for environmentally friendly technologies and products has contributed to the wider use of heterogeneous catalysis [17,18,19]. This is primarily due to the possibility of regeneration of the catalyst. It also eliminates the need to neutralize the reaction mass, rinse the final product and, accordingly, does not lead to pollution emissions [20,21,22].

From the literature, it is known to use a wide range of heterogeneous amphoteric catalysts, including zinc oxide [23,24,25].

Zinc oxide is an inexpensive, affordable, environmentally friendly catalyst that allows the esterification of carboxylic acids with alcohols in the absence of solvent [26,27,28,29,30,31,32].

In the production of PVC plasticates, zinc compounds are used as stabilizing additives [33,34]. The low thermal stability of polyvinyl chloride leads to the loss of HCl at a temperature above 100 °C, which further contributes to the formation of colored and easily oxidizable conjugated polyene structures, to chain breaks and, accordingly, to the deterioration of mechanical properties [35].

Among a wide range of zinc-containing stabilizing additives, the use of zinc carboxylates (e.g., zinc stearate) is known [36]. The effect of these compounds is explained by the Frye–Horst mechanism [37,38], which proved that stabilization results from the exchange of labile chlorine atoms for more stable carboxylate groups.

The metal soaps most commonly used to stabilize PVC compositions are prone to dust formation and are, therefore, unsafe from the point of view of industrial hygiene. Therefore, complex functional additives are required to optimize the production of PVC composites.

As a result of the above comments, the goal of this work is the synthesis of a new ester plasticizer of polyvinyl chloride based on adipic acid ester using an environmentally friendly zinc oxide catalyst and the development of a plasticizing composition containing a stabilizing additive obtained from zinc oxide in situ.

## 2. Materials and Methods

### 2.1. Starting Materials

Adipic acid (Radici Group, Selbitz—Hochfranken, Bavaria, Germany) is a white crystalline substance with a main substance content of 99.8%, and was used as received; it has a melting point (m.p.) of 153 °C. Butyl alcohol (Salavatnefteorgsintez, Salavat, Russia) is a colorless transparent flammable liquid that has a peculiar smell, with a main substance content of 99.4%, is poorly soluble in water, and mixes with alcohol and other organic solvents with a boiling point (m.b.) of 117.4 °C; it was used as received. Phenol (“Ufaorgsintez,” Ufa, Russia) is a colorless needle-like crystal, and was used as received; it has a melting point (m.p.) of 40.9 °C. Ethylene oxide (ECOTECH Chemical Components Plant, Moscow, Russia) is a liquefied gas that is a colorless transparent liquid in steel cylinders with a boiling point (m.b.) of 10.7 °C. Sodium hydroxide (Joint Stock Company “Caustic,” Sterlitamak, Russia) is a white solid with a main substance content of 98.2%, and was used as received. Zinc oxide (Component-Reagent, Moscow, Russia) is a colorless crystalline powder with a main substance content of 98%, is insoluble in water, and yellows when heated. Toluene (Public Joint-Stock Company “Joint-Stock Oil Company Bashneft,” Ufa, Russia) is a colorless liquid with a characteristic smell a main substance content of 99%, and was used as received; it has a boiling point (b.p.) of 110.6 °C. Polyvinyl chloride (Joint Stock Company “Caustic,” Sterlitamak, Russia): We used industrial samples of suspension polyvinyl chloride PVC 7059M.

### 2.2. Synthesis Methods

#### 2.2.1. Synthesis of Butoxyethanol

A round-bottom flask equipped with a thermometer, a stirrer, a reflux condenser, and a device for injecting ethylene oxide into the reaction mass was filled with 74 g (1 mol) of butanol and a sodium hydroxide catalyst in an amount of 1.1 g (1% by weight).

The reactor was heated to 110–180 °C and purged with nitrogen to remove air. Subsequently, 44 g (1 mol) of ethylene oxide was gradually injected to the operation, which has a stirrer. The ethylene oxide feed rate was adjusted so that the unreacted oxide condenses in the cooler and returns to the reactor without flooding. After feeding the entire amount of ethylene oxide, the temperature of the reaction mixture was maintained for an additional 1–1.5 h and then cooled to room temperature.

Catalyst was neutralized with a calculated amount of sulfuric acid, and the obtained mass was filtered. The reaction mixture was then distilled off fraction boiling to 50 °C at 10 mm Hg.

Butoxyethanol is a colorless oily liquid soluble in water. The yield was 106.8 g (90.5% of theoretical).

#### 2.2.2. Synthesis of Phenoxyethanol

A round-bottom flask equipped with a thermometer, a stirrer, a reflux condenser, and a device for introducing ethylene oxide into the reaction mass was charged with 94 g (1 mol) of phenol and a sodium hydroxide catalyst in an amount of 1.4 g (1% by weight).

The reactor was heated to 110–180 °C and purged with nitrogen to remove air. Subsequently, 44 g (1 mol) of ethylene oxide was gradually introduced with the stirrer in operation. The ethylene oxide feed rate was adjusted so that the unreacted oxide condenses in the cooler and returns to the reactor without flooding. After feeding the entire amount of ethylene oxide, the temperature of the reaction mixture was maintained for an additional 1–1.5 h and then cooled to room temperature.

Catalyst was neutralized with a calculated amount of sulfuric acid, and the obtained mass was filtered. The reaction mixture was then distilled off fraction boiling to 50 °C at 10 mm Hg.

Phenoxyethanol is a colorless oily liquid soluble in water. The yield was 122.8 g (89% of theoretical).

#### 2.2.3. The synthesis of Butoxyethyl Phenoxyethyl Adipate (BEPEA)

First, 150 mL of toluene and 138 g (1 mol) of phenoxyethanol were charged into the reaction flask and stirred until the reagents were completely dissolved. Then, 2.8 g (1% by weight) of zinc oxide and 146 g (1 mol) of adipic acid were added, and the temperature was slowly raised to 110 °C. Stirring was continued for 1.5 h. The end of the reaction was determined by the amount of released water.

Then, without isolating the monoester, it was esterified with butoxyethanol (110 g) taken in the molar ratio monoester: Alcohol = 1:0.93. Boiling was continued for 2 h. The reaction mixture was cooled, and the product was filtered and dried at 125 °C. The yield of the light powder product was 320.8 g (89% of theoretical). The product was a mixture of butoxyethyl phenoxyethyl adipate (302.6 g) plasticizer and zinc phenoxyethyl adipate stabilizer in an amount of 18.2 g.

Characteristics of the obtained products are presented hereinafter.

### 2.3. Methods of Analysis

The analysis of physicochemical parameters of the obtained compounds was carried out in accordance with state standard 8728-88 Plasticizers. Specifications [39]. For this, the following indicators were determined: Acid number, ester number, mass fraction of volatile substances.

Analysis of the acid number. The essence of the definition is the titration of an alcoholic solution of the test product with a solution of potassium hydroxide in the presence of a phenol red indicator. The acid number (X) in mg KOH/g is calculated by Formula (1):

(1) $$X=\frac{V\;\cdot \;5.61}{m}\;$$

where V —volume of 0.1 N sodium hydroxide solution used for titration of the sample, cm^3^; 5.61—equivalent weight of potassium hydroxide; m —sample weight taken for analysis, g.

The analysis of the ester number. The essence of the definition consists of titration with a solution of hydrochloric or sulfuric acid in the presence of phenolphthalein until the sample of a plasticizer and the solution of potassium hydroxide are discolored after heating for 1 h in a boiling water bath. The ester number (X) in mg KOH/g is calculated by Formula (2):

(2) $$X_{1}=\frac{(V_{1}-V_{2})\;\cdot \;56.1}{m}$$

where $V_{1}$—the volume of a solution of hydrochloric or sulfuric acid with a concentration of 1 mol/dm^3^ (1 n), used for titration in a control experiment, cm^3^; $V_{2}$—the volume of a solution of hydrochloric or sulfuric acid with a concentration of 1 mol/dm^3^ (1 n) spent on titration of a solution with an analyzed sample, cm^3^; 56.1—equivalent weight of potassium hydroxide; m —sample weight taken for analysis, g.

Density was determined according to state standard 18329–2014 Liquid resins and plasticizers. Methods for determination of density [40].

The refractive index was determined on an IRF-22 refractometer according to state standard 18995.2-73 Liquid chemical products. Method for determination of refractive index [41].

For thermogravimetric analysis, a TGA/DSC-1 device (Mettler Toledo company, Greifensee, Switzerland) was used. The temperature range of the device was 25–1100 °C. The maximum volume of the test sample did not exceed 900 μL. The maximum heating rate of the sample was 150 K/min. The cooling rate of the device was 20 K/min. Measurement accuracy ± 0.3 K.

A sample of the test sample (5–15 mg) was placed in an aluminum crucible with a volume of 70 μL. The measurements were carried out in the temperature range from 25 °C to 500 °C at different heating rates, with atmosphere—air, nitrogen. Processing of the results was carried out using a computer. A curve was displayed on the computer screen as a function of the mass of the sample versus temperature (TGA curve), as well as a DSC curve characterizing the thermal effects that occur during the destruction of the test sample.

Determination of the color stability of the PVC composition at 180 °C (state standard 14041–91) [42]. The test PVC material is placed in a test tube so that it is 50 mm full. A second tube is also filled. Shake each tube carefully so that the pieces do not form a compact mass or stick to the walls of the tube. The oil bath is preheated to a temperature close to the specified test temperature (180 °C for plasticized compositions and products), and the temperature is then adjusted to the exact temperature indicated on the thermometer. For each tube, a strip of test paper 30 mm long and 10 mm wide is cut or selected. From one end, a strip of indicator paper is folded or twisted and inserted into a glass tube. Wet a strip of indicator paper with double-distilled water. The glass tube is inserted into the cork. The tube with the stopper is inserted into the tube, the tube is closed with the stopper, and the position of the glass tube is adjusted in the stopper so that the bottom edge of the paper is 25 mm above the surface of the sample. The tube is immersed in an oil bath to the level of the upper surface of the test sample. The stopwatch measures the time until the first noticeable color change from red to blue appears on the Congo red indicator paper. When using universal indicator paper, the end point should correspond to a color that characterizes PH = 3.

Determination of thermostability of PVC compound at 160 °C (state standard 14332–78) [43]. Prepare a mixture consisting of 100 parts-by-weight polyvinyl chloride, 50 parts-by-weight plasticizer, and 0.305 parts-by-weight cadmium stearate, stirred in a glass with a spatula until smooth and heated for 30–40 min in a water bath at 80–90 °C. The prepared mixture is loaded onto rollers preheated to (160 ± 2) °C, and a film is rolled (2 ± 0.2) mm thick for 2–4 min with a roll gap of 0.4–0.5 mm. Then, the gap is adjusted in accordance with the thickness and rolled for 5 min. Then, 8–10 samples of size 6 × 40 × (2 ± 0.2) mm are cut from the film. The tubes are filled 50 mm in height with liquid paraffin and placed in a thermostat for 15 min at (160 ± 1) °C, making sure that the oil level in the bath is approximately 10 mm higher than the oil level in the tube. Then, one sample is simultaneously immersed in each tube. Samples are taken out sequentially every 5 min, cooled and compared with each other, and pre-attached to the chart indicating the test time. For the test result, the maximum time in minutes is taken, for which the color of the test sample has not changed compared to the control sample.

Determination of indicators of tensile stress and elongation at break (state standard 9998–86) [44]. Samples are secured to the clamps of the testing machine. They are uniformly tightened so that the specimen does not slip during testing, but the specimen is not destroyed at the fixing point. Tests are carried out at a temperature of (23 ± 2) °C; and relative humidity (50 ± 5)%.

Tensile strength (MPa) is calculated by the Formula (3):

(3) $$\sigma_{z}=\frac{F_{max}}{A_{0}}$$

where $F_{max}$—maximum tensile load during tensile test, N; $A_{0}$—initial cross-section of the sample, mm^2^.

Elongation (%) is calculated by Formula (4):

(4) $$\varepsilon_{r}=\frac{\Delta l_{0r}}{l_{0}}\;\cdot 100$$

where $l_{0}$—initial calculated sample length, mm; $\Delta l_{0r}$—change in the estimated length of the sample at the time of rupture, mm.

## 3. Results and Discussion

### 3.1. Synthesis of Ethoxylated Alcohols

**Scheme 1:**
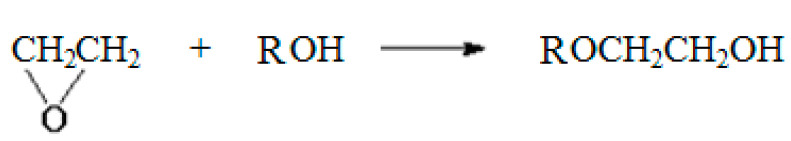
Synthesis of ethoxylated alcohols.

The oxyethylation reaction of alcohols has been well studied and carried out on an industrial scale [45,46]. The ethoxylation of alcohols was carried out according to well-known methods by their reaction with ethylene oxide at a temperature of 110–180 °C (Scheme 1). Sodium hydroxide was used as a catalyst. The chemistry and mechanism of this reaction are well studied [47]. The characteristics of the products obtained are given in Table 1.

where R—Bu, Ph.

### 3.2. The Synthesis of Butoxyethyl Phenoxyethyl Adipate (BEPEA)

In previous studies, the preparation of adipic acid esters by esterification using homogeneous catalysis was described, and their physicochemical properties for plasticization of polyvinyl chloride were studied [9,14,15,48]. This article discusses a method for the synthesis of a new ester compound of butoxyethyl phenoxyethyl adipate (BEPEA) using a heterogeneous zinc oxide catalyst.

This catalyst has been selected for several reasons: First, in order to improve the method of producing adipate plasticizer. The use of zinc oxide in esterification avoids additional steps of neutralization and purification of the resulting product. Secondly, to improve the quality of the resulting product. Ester plasticizers in this case are more pure, which corresponds to an increase in the yield of the desired product. In addition, the purpose of our work was to obtain zinc carboxylate together with the new BEPEA plasticizer, as it is known that zinc compounds are used as stabilizers for PVC compositions.

**Scheme 2:**
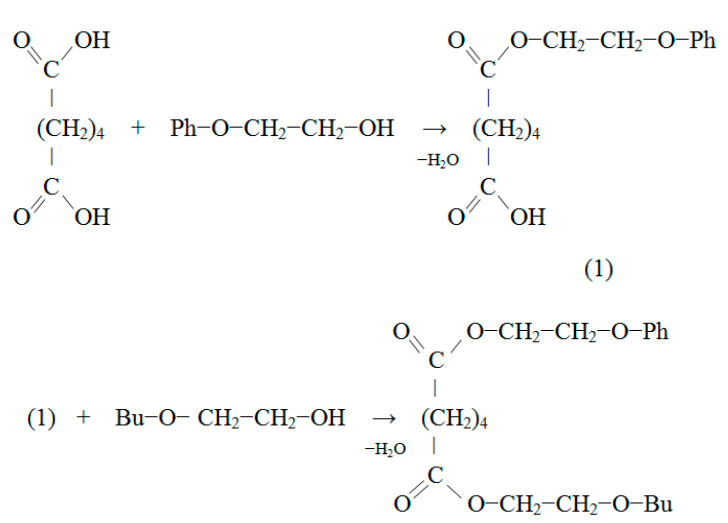
Synthesis of butoxyethyl phenoxyethyl adipate (BEPEA).

The target ester was obtained in two stages (Scheme 2).

In the first stage, an adipic acid monoester was prepared by reacting ethoxylated phenol with adipic acid, taken in an equimolar ratio, in the presence of an azeotropic water-carrying agent toluene and zinc oxide catalyst in an amount of 1 wt%. from total component loading. Then, without isolating the monoester, it was esterified with the calculated amount of butoxyethanol. The end of the reaction was determined by the amount of released water and the acid number of the esterificate. The reaction mixture was cooled, and the product was filtered and dried at 125 °C. As a result, adipic acid ester and zinc phenoxyethyl adipate were obtained together.

For research, the bulk of the obtained product was thoroughly washed from the zinc compound and pure ester was obtained, which was further confirmed on a DSC thermogram. The remainder of the ester was used for analysis together with the zinc compound in the form of a plasticizing composition. Physicochemical properties of the obtained adipic acid ester are shown in Table 2.

To study the possibility of practical use of a plasticizer in PVC composites, it is important to meet the modern requirements of the following indicators: Thermal stability, melting and crystallization temperatures, compatibility with the polymer.

**Figure 1:**
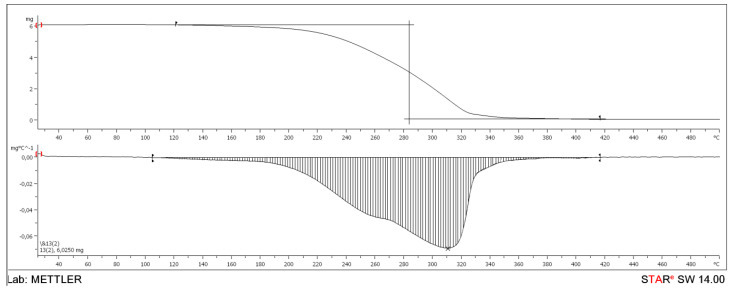
The thermal stability of the plasticizer of BEPEA by thermogravimetry, using a TGA-DSC-combined thermal analysis instrument.

The thermal stability of the plasticizers was studied by thermogravimetry using a TGA-DSC-combined thermal analysis instrument (Mettler Toledo) in the temperature range of 20–500 °C at a heating rate of 5 °C/min (Figure 1). For comparison, a sample of industrial plasticizer di-2-ethylhexylphthalate (DOP) is usually used.

To this end, weight reduction was also evaluated by heating the plasticizer to temperatures of 180 °C and 200 °C corresponding to the processing temperature interval of PVC compositions (Δm) (Table 3).

From the results of thermal analysis, it follows that the decomposition of the studied BEPEA plasticizer occurs in the temperature range from 121 to 417 °C (Table 3). The obtained plasticizer is characterized by a high temperature T_s_—121 °C, which is somewhat inferior to the same parameter for industrial DOP (Table 3).

It should also be noted that the parameter Δm is relatively low for the obtained BEPEA plasticizer at temperatures of 180 and 200 °C, which characterizes the content of volatile impurities in the product that can be released during the processing of the plasticized PVC composition at the molding of materials and products. Thus, for the BEPEA plasticizer, the obtained Δm value at 180 °C is 1.1%, compared to industrial DOP—1.0% (Table 3).

Thus, the BEPEA plasticizer in terms of thermal stability has values close to the industrial plasticizer DOP.

**Figure 2:**
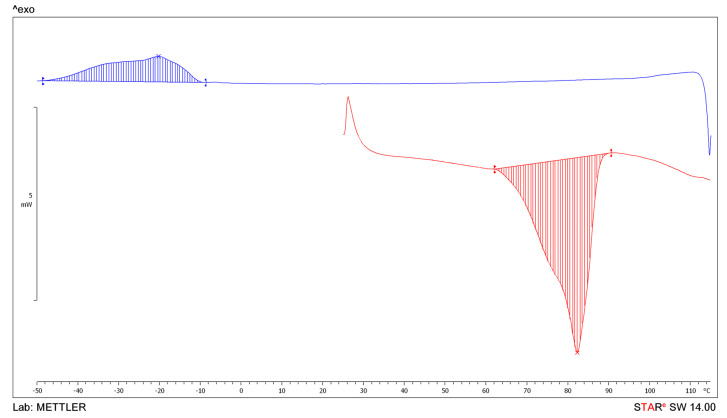
DSC-thermogram of a plasticizer of BEPEA.

In contrast to DOP, the synthesized plasticizer is a solid product; therefore, melting and crystallization temperatures are important for its practical use. These indicators, as well as the enthalpy of these processes, were determined by differential scanning calorimetry on a DSC-1 instrument (Mettler Toledo) in the temperature range from −50 to 115 °C at a heating/cooling rate of 5 °C/min (Figure 2).

An endothermic peak corresponding to the melting process with a maximum of T_m_ = 82.3 °C is fixed on the thermogram of the plasticizer in the heating mode in the temperature range of 62–91 °C. When the sample is cooled in the temperature range of (−49)–(−8) °C, there is an exothermic peak of low intensity with a maximum of T_cr_ = −20 °C.

Values of the enthalpy of melting ΔH_m_ and crystallization ΔH_cr_ corresponding to the indicated peaks in the DSC thermogram are given in Table 4.

An important parameter determining the possibility of using a plasticizer in polymer compositions is its compatibility with the polymer. One of the methods for determining the compatibility of a plasticizer with PVC is an assessment of the critical temperature of polymer dissolution in a plasticizer using a critical temperature T_c_.

It was found that the dissolution temperature of PVC in the BEPEA plasticizer is noticeably (by 59 °C) higher than that in DOP (Table 5), which indicates the lower compatibility of this plasticizer with the polymer. Apparently, the obtained compound can be classified as secondary plasticizers, which are partially compatible with the polymer and used as a mixture with a primary plasticizer, for example, with DOP [1,2,4].

Therefore, a mixture of DOP with synthesized plasticizer BEPEA is in the ratio of 5:1 (wt.). The compatibility of PVC with this mixture of plasticizers (T_c_ = 119 °C) is slightly lower than with DOP (Table 5); however, this mixture can be used as part of PVC composites.

Then, to assess the effect of the zinc compound in the composition of the obtained butoxyethyl phenoxyethyl adipate plasticizer on the stabilization of PVC, the following compositions were prepared:

- **1**—basic formulation (PVC 7059M—100 parts by mass, plasticizer DOP—42 parts by mass, stabilizer calcium stearate—3 parts by mass)
- **2**—basic formulation containing 42 parts by weight of a mixture of plasticizers DOP:BEPEA in a ratio of 35:7
- **3**—basic formulation containing, instead of the BEPEA plasticizer, a plasticizing composition with a zinc compound.

The resulting films were evaluated by processability indicators and operational characteristics, the data on which are given in Table 6. Physicomechanical tests of PVC films were carried out according to state standard 9998-86. The indicators of color and thermal stability were evaluated in accordance with state standard 14041-91 and state standard 14332-78, respectively.

It can be seen from the obtained results that the increase in color and thermal stability for composition **2** in comparison to the similar data for composition **1** is probably due to the synergy of the developed plasticizer BEPEA and DOP.

The best indicators of color and thermal stability are characteristic for composition **3** due to the combination of zinc compounds and calcium stearate, acting on various stabilization mechanisms. In particular, the zinc compound stabilizes PVC by the nucleophilic mechanism, replacing the labile chlorine atoms in the PVC macromolecules, and calcium stearate binds hydrogen chloride, a result of which the zinc compound is regenerated.

Physico-mechanical properties of PVC compositions with the content of the developed plasticizer and the developed plasticizing mixture correspond to the normative.

Thus, a promising composition is formulation 3 containing a mixture of plasticizers DOP and butoxyethyl phenoxyethyl adipate in a weight ratio of 5:1 and a zinc compound in small quantities.

## 4. Conclusions

The reaction of esterification of adipic acid with ethoxylated alcohols in the presence of a heterogeneous zinc oxide catalyst produced a new environmentally safe plasticizing composition containing 94.3% butoxyethyl phenoxyethyl adipate plasticizer and 5.7% zinc phenoxyethyladipate stabilizing additive. The yield of the plasticizing composition was 89%.

The physical and chemical properties of butoxyethyl phenoxyethyl adipate plasticizer, as well as its physical and mechanical characteristics for studying the possibility of practical use in PVC composites, were studied. It was revealed that the ester developed is limited to the compatibility with the PVC polymer in plasticate, so studies were carried out in a mixture with DOF in the ratio of DOF:BEPEA = 5:1.

The test results of the developed PVC composition showed that partial replacement of DOF with the developed plasticizer butoxyethyl phenoxyethyl adipate in the base formulation increases color stability by 20% and thermal stability by 6%. The physical and mechanical characteristics of the obtained compositions meet the normative requirements.

The best results are obtained by replacing the BEPEA plasticizer with the developed plasticizing composition. Thus, color stability increases by 80%, and thermal stability by 21%.

The resulting PVC composition is very promising, as the plasticizer included in the composition contributes to the expansion of the range of non-toxic alternatives to widely used phthalates, and the stabilizing additive is an environmentally safe zinc compound.

## Figures and Tables

**Table 1:** Physico-chemical properties of ethoxylated alcohols.

Indicators	Butoxyethanol	Phenoxyethanol
Density, d^20^_4_	0.9648	1.1007
Refractive index, n ^20^_d_	1.4267	1.5314
Molecular weight	118	138

**Table 2:** Physicochemical properties of butoxyethyl phenoxyethyl adipate (BEPEA).

Ester	Indicators			
	Molecular Weight	Acid Number, mg KOH/g	Ester Number, mg KOH/g	d^20^_4_
BEPEA	407	0.1	274	1.0042

**Table 3:** The results of the thermal analysis of plasticizers.

Sample	Characteristics				
	Temperature, °C			Δm, %	
	Beginning	Maximal Value	End	at 180 °C	at 200 °C
BEPEA	121.47	310.67	417.16	1.1	1.9
DOP	134.07	284.83	497.89	1.0	1.9

**Table 4:** DSC analysis of butoxyethylphenoxyethyl adipate plasticizer.

Sample	Endotherm Characteristics				Exotherm Characteristics			
	Temperature, °C			ΔH_m_, J/g	Temperature, °C			ΔH_cr_, J/g
	Beginning	Maximal Value	End		Beginning	Maximal Value	End	
BEPEA	73.89	82.33	87.42	−66.4	−10.58	−20.17	−44.35	34.4

**Table 5:** The results of determining the critical temperature of the dissolution of plasticizers.

Plasticizer	T_c_, °C
DOP	113
BEPEA	172
Mixture DOP + BEPEA	119

**Table 6:** PVC composition test results.

Name of Indicator	PVC Composition		
	1	2	3
Tensile stress, MPa	16.1	14.0	14.2
Elongation at break,%	225	232	234
Color stability at 180 °C, min	25	30	45
Thermostability at 160 °C, min	80	85	97
